# Supplementary material for: Iterative improvement in the automatic modular design of robot swarms
Source: PeerJ Comput Sci. 2020 Dec 7;6:e322. doi: 10.7717/peerj-cs.322 (PMC7924708; doi:10.7717/peerj-cs.322)
Supplement: Supplemental Information 3 [file peerj-cs-06-322-s003.zip › argos3/doc/api/standalone/a00492_source.html]

ARGoS: plugins/robots/generic/control\_interface/ci\_differential\_steering\_actuator.cpp Source File


- Main Page
- Related Pages
- Namespaces
- Classes
- Files

- File List
- File Members

# plugins/robots/generic/control\_interface/ci\_differential\_steering\_actuator.cpp

Go to the documentation of this file.

```
00001 
00007 #include "ci_differential_steering_actuator.h"
00008 
00009 #ifdef ARGOS_WITH_LUA
00010 #include <argos3/core/wrappers/lua/lua_utility.h>
00011 #endif
00012 
00013 namespace argos {
00014 
00015    /****************************************/
00016    /****************************************/
00017 
00018 #ifdef ARGOS_WITH_LUA
00019    /*
00020     * The stack must have two values in this order:
00021     * 1. left wheel speed (a number)
00022     * 2. right wheel speed (a number)
00023     */
00024    int LuaSetDiffSteeringLinearVelocity(lua_State* pt_lua_state) {
00025       /* Check parameters */
00026       if(lua_gettop(pt_lua_state) != 2) {
00027          return luaL_error(pt_lua_state, "robot.wheels.set_velocity() expects 2 arguments");
00028       }
00029       luaL_checktype(pt_lua_state, 1, LUA_TNUMBER);
00030       luaL_checktype(pt_lua_state, 2, LUA_TNUMBER);
00031       /* Perform action */
00032       CLuaUtility::GetDeviceInstance<CCI_DifferentialSteeringActuator>(pt_lua_state, "wheels")->
00033          SetLinearVelocity(lua_tonumber(pt_lua_state, 1),
00034                            lua_tonumber(pt_lua_state, 2));
00035       return 0;
00036    }
00037 #endif
00038    
00039    /****************************************/
00040    /****************************************/
00041 
00042 #ifdef ARGOS_WITH_LUA
00043    void CCI_DifferentialSteeringActuator::CreateLuaState(lua_State* pt_lua_state) {
00044       CLuaUtility::OpenRobotStateTable(pt_lua_state, "wheels");
00045       CLuaUtility::AddToTable(pt_lua_state, "_instance", this);
00046       CLuaUtility::AddToTable(pt_lua_state, "set_velocity", &LuaSetDiffSteeringLinearVelocity);
00047       CLuaUtility::CloseRobotStateTable(pt_lua_state);
00048    }
00049 #endif
00050 
00051    /****************************************/
00052    /****************************************/
00053 
00054 }
```

---

Generated on 10 Jul 2018 for ARGoS by 
 1.6.1
